# Co-Medication of Statins with Contraindicated Drugs

**DOI:** 10.1371/journal.pone.0125180

**Published:** 2015-05-01

**Authors:** Bo Ram Yang, Jong-Mi Seong, Nam-Kyong Choi, Ju-Young Shin, Joongyub Lee, Ye-Jee Kim, Mi-Sook Kim, Soyoung Park, Hong Ji Song, Byung-Joo Park

**Affiliations:** 1 Department of Preventive Medicine, Seoul National University College of Medicine, Seoul, Republic of Korea; 2 Medical Research Collaborating Center, Seoul National University Hospital, Seoul, Republic of Korea; 3 Korea Institute of Drug Safety and Risk Management, Seoul, Republic of Korea; 4 Department of Family Medicine, Hallym University College of Medicine, Chuncheon, Korea; Universitätsklinikum des Saarlandes, GERMANY

## Abstract

**Background:**

The concomitant use of cytochrome P450 3A4 (CYP3A4) metabolized statins (simvastatin, lovastatin, and atorvastatin) with CYP3A4 inhibitors has been shown to increase the rate of adverse events.

**Objective:**

This study was performed to describe the co-medication prevalence of CYP3A4-metabolized statins with contraindicated drugs.

**Methods:**

The patients aged 40 or older receiving CYP3A4-metabolized statin prescriptions in 2009 were identified using the national patient sample from a Korea Health Insurance Review and Assessment Service database. Contraindicated co-medication was defined as prescription periods of statins and contraindicated drugs overlapping by at least one day. Co-medication patterns were classified into 3 categories as follows: co-medication in the same prescription, co-medication by the same medical institution, and co-medication by different medical institutions. The proportion of co-medication was analyzed by age, gender, co-morbidities, and the statin’s generic name.

**Results:**

A total of 2,119,401 patients received CYP3A4-metabolized statins and 60,254 (2.84%) patients were co-medicated with contraindicated drugs. The proportion of co-medication was 4.6%, 2.2%, and 1.8% in simvastatin, lovastatin, and atorvastatin users, respectively. The most frequent combination was atorvastatin-itraconazole, followed by simvastatin-clarithromycin and simvastatin-itraconazole. Among the co-medicated patients, 85.3% were prescribed two drugs by different medical institutions.

**Conclusion:**

The proportion of co-medication of statins with contraindicated drugs was relatively lower than that of previous studies; however, the co-medication occurring by different medical institutions was not managed appropriately. There is a need to develop an effective system and to conduct outcomes research confirming the association between co-medication and the risk of unfavorable clinical outcomes.

## Introduction

Hydroxymethylglutaryl-CoA (HMG-CoA) reductase inhibitors (statins) are considered to be the first-choice drug for patients with dyslipidemia to lower their cholesterol levels. Several studies have reported the effects of statins on primary and secondary prevention of cardiovascular events and mortality [1–6]. Although, statins are generally considered to be well tolerated and to have a good safety profile, statin-induced myopathy including rhabdomyolysis has been reported [7]. Cerivastatin was withdrawn from the market as a result of a high frequency of myopathy and rhabdomyolysis in 2001. The exact mechanisms of statin-induced myopathy have not been fully determined. However, drug-drug interaction (DDI) is considered to be a risk factor for this adverse effect of statins [8]. Previous studies also support the importance of DDI among statin users. Data from spontaneous adverse event reports indicated that approximately 50% of rhabdomyolysis cases among statin users are associated with DDIs [9].

The difference in the drug interaction potential between individual statins is known. The DDI potential of cytochrome P450 3A4 (CYP3A4)-metabolized statins (simvastatin, lovastatin, and atorvastatin) is higher than for other statins, because many agents act as inhibitors or inducers for CYP3A4. Concomitant use of potent CYP3A4 inhibitors such as anti-bacterial macrolides, anti-fungal agents, and protease inhibitors reduces the pre-systemic metabolism of CYP3A4-metabolized statins and increases statins’ plasma concentration [10,11]. This drug interaction can lead to rhabdomyolysis, which is the most serious type of statin induced myopathy, acute kidney injury, and death [12]. The relative risk of hospitalization for myopathy in patients who received statins with concomitant use of CYP3A4 inhibitors has been found to be similar to that in patients with cerivastatin therapy [13]. In Korea, these strong DDIs were deemed contraindicated drugs by the Korea Ministry of Health and Welfare (KMHW) and co-medication of contraindicated drugs has been managed by a national Drug Utilization Review system.

It is necessary to evaluate the status of co-medication of CYP3A4-metabolized statins with contraindicated drugs. No published study focusing on co-medication has specifically distinguished among co-prescription, co-medication occurring by the same medical institution, and that by different medical institutions. Therefore, we aimed to investigate the co-medication of CYP3A4-metabolized statins with contraindicated drugs.

## Materials and Methods

### Data source and study subjects

We used the 2009 national patient sample from the Korea Health Insurance Review and Assessment Service database (HIRA-NPS). The National Health Insurance (NHI) program was initiated in Korea in 1977 and achieved universal coverage of the entire Korean population by 1989. Accordingly, the HIRA database contains all medical information for approximately 50 million Koreans. National patient samples of the HIRA database were constructed by a stratified random sampling method for age interval of 5 year and gender from patients visiting healthcare institutions in 2009 [14]. The HIRA-NPS database contained information on 1,116,040 patients representing total 45,969,893 patients. The HIRA-NPS database included the inpatient sample (711,457) which was 13% of the entire hospitalized patients (n = 5,472,670), and outpatient sample (404,583) which was 1% of entire outpatient population.

The HIRA-NPS database contained each patient’s unique encrypted identification number (ID), age, gender, the prescription number, and the medical institution’s identifier. In addition, prescribed drug information included the generic name, brand name, prescription date, dose, duration, and route of administration. The diagnosis was coded according to the International Classification of Disease, Tenth Revision (ICD-10).

We performed a population-based cross-sectional study. The study population consisted of all patients aged 40–99 years who received at least one prescription of any CYP3A4-metabolized statin (simvastatin, atorvastatin, and lovastatin) in 2009.

### Drug exposure assessment

The exposure of CYP3A4-metabolized statins and contraindicated combinations was assessed in the study subjects. Contraindicated combinations consisted of anti-bacterial macrolides (erythromycin and clarithromycin), anti-fungal agents (ketoconazole and itraconazole) and protease inhibitors (indinavir, nelfinavir, ritonavir, lopinavir/ritonavir, and atazanavir), which were included as potent CYP3A4 inhibitors. Topical agents were not included in the list of contraindicated drugs (Table 1). The medications with a contraindication due to DDI were selected on the basis of Notification No. 2004–2 and Notification No. 2005–17 issued by the KMHW, and Notification No.2007-68 issued by Ministry of Food and Drug Safety (MFDS) [15–17]. Since 2004, the KMHW and MFDS have issued the Contraindicated-combination Drug List, which addressed drug-drug interactions. When each drug was labeled as a contraindicated medication, the MFDS noted the drug pair as contraindicated; they were then reviewed in a drug utilization review sub-committee in accordance with the literature and reference databases.

**Table 1:** List of statins and contraindicated drugs in Korea.

Medication	Contraindicated drugs	Therapeutic class
Simvastatin	Erythromycin, Clarithromycin,	Anti-bacterial macrolides
	Ketoconazole, Itraconazole	Anti-fungal agents
	Indinavir, Nelfinavir, Ritonavir, Lopinavir + Ritonavir, Atazanavir	Protease inhibitors
Atorvastatin	Ketoconazole	Anti-fungal agents
	Itraconazole	
Lovastatin	Ketoconazole, Itraconazole	Anti-fungal agents
	Atazanavir	Protease inhibitors

**Fig 1:**
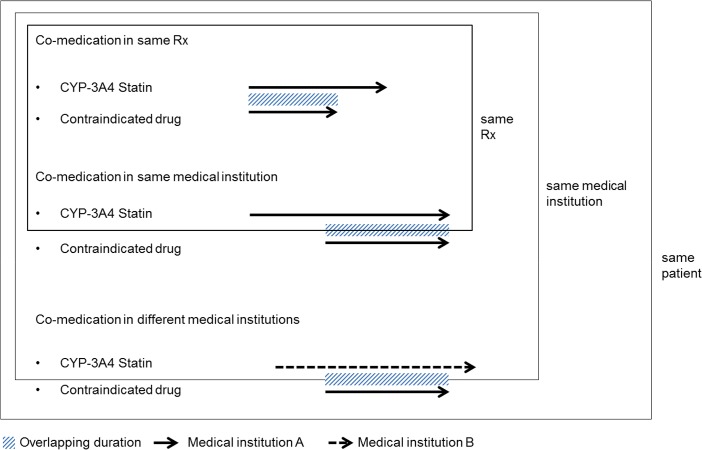
Classification of co-medication patterns. Rx = Prescription.

Firstly, we identified CYP3A4-metabolized statins user during 2009, and then constructed database that contained all CYP3A4-metabolized statins or contraindicated medications use included generic name, prescription date, and duration. Contraindicated co-medication was defined as overlapping of prescription periods of statins and contraindicated drugs by at least one day in a given patient using each drug’s prescription date, duration, unique prescription number, and medical institution’s identifiers. We classified the patterns of co-medication as one of three types as follows: 1) co-medication in the same prescription, 2) co-medication by the same medical institution, and 3) co-medication by different medical institutions. Co-medication in the same prescription was defined as prescribing of a statin and contraindicated drug in one prescription which was confirmed by unique prescription number. Co-medication in the same prescription was defined as prescribing of a statin and contraindicated drug in one prescription. Co-medication by the same medical institution was defined as prescriptions of a statin and a contraindicated drug by one medical institution with overlapping prescription periods, but different prescriptions. Co-medication by different medical institutions was defined as prescriptions from separate medical institutions with overlapping prescription periods [18] (Fig 1).

### Statistical analysis

Statin users were grouped by the prescribed statin’s generic name. If a patient switched to another CYP3A4-metabolized statin or received more than one CYP3A4-metabolized statin, the patient could contribute to more than one group. Patients who were co-medicated with contraindicated drugs were identified among CYP3A4-metabolized statin users. The proportion of co-medicated patients was calculated according to gender, age, the statin’s generic name, and co-morbidities with weighted analysis. Continuous variables were described using means, and their standard error (SE), and categorical variables were described using percentages and SE. To compare the proportions of each gender, the statin’s generic name, and co-morbidities, the chi-squared test was used. The significance level was set at a *p*-value < 0.01.

The frequency and proportion of co-medication were estimated according to the statin’s generic name. The co-prescription was identified using the unique prescription number. The co-medication in the same medical institution was identified by assessing the unique prescription number, patient’s encrypted identification ID, and medical institution’s identifier. Some patients identified with more than one co-medication pattern were counted multiple times in the corresponding groups.

For the CYP3A4-metabolized statin users with any exposure to contraindicated drugs, the duration of concomitant exposure was described. The overlapping period of CYP3A4-metabolized statin and contraindicated drugs was presented by the statin’s generic name and co-medication pattern using the mean (±SE) and median (Q1-Q3) days of overlap.

SAS for Windows version 9.3 (SAS Institute Inc., Cary, NC, USA) was used to perform all statistical analyses.

### Ethics statement

The study protocol was approved by the institutional review board of the Seoul National University College of Medicine (IRB No. 1001-023-306). Obtaining informed consent from the study population was waived by the board.

## Results

### Patient demographics and co-medication according to characteristics

A total of 2,119,401 patients who were prescribed CYP3A4-metabolized statins were included in the analyses from the HIRA database in 2009. Overall, 59.8% were female and the mean age (±SE) was 60.8 (±0.1) years (Table 2). The majority of patients received atorvastatin (71.6%), followed by simvastatin (32.8%), and then by lovastatin (2.8%).

**Table 2:** The proportion of patients co-medicated with statins and contraindicated drugs.

Characteristics		Total statin users		Co-medicated patients			*p-*value^*^
		Number	(%)	Number	%	(SE)	
Total		2,119,401	(100.0)	60,254	2.84	0.10	
Gender							<.01
Male		852,841	(40.24)	23,953	2.81	0.16	
Female		1,266,560	(59.76)	36,300	2.87	0.13	
Age	(Mean±SE)	(60.8±0.1)		(60.4±0.3)			0.01
40–44		120,285	(5.68)	2,285	1.90	0.34	
45–49		212,950	(10.05)	5,823	2.73	0.31	
50–54		330,059	(15.57)	9,816	2.97	0.26	
55–59		327,915	(15.47)	10,454	3.19	0.28	
60–64		322,379	(15.21)	10,607	3.29	0.28	
65–69		327,222	(15.44)	10,031	3.07	0.25	
70–74		246,205	(11.62)	6,477	2.63	0.25	
75+		232,386	(10.96)	4,761	2.05	0.24	
Statin use							<.01
Simvastatin		695,941	(32.84)	32,085	4.61	0.22	
Atorvastatin		1,517,021	(71.58)	27,354	1.80	0.09	
Lovastatin		59,861	(2.82)	1,300	2.17	0.55	
Comorbidities							
Dyslipidemia		2,075,847	(97.94)	59,454	2.86	0.10	0.15
Hypertension		1,504,981	(71.01)	44,600	2.96	0.12	0.06
Diabetes mellitus		918,894	(43.36)	28,654	3.12	0.15	0.01
Ischemic heart disease		521,127	(24.59)	16,184	3.11	0.19	0.26
Other vascular disease		490,911	(23.16)	14,892	3.03	0.20	0.12

SE = Standard error

*p-value was estimated by chi-square test.

**Fig 2:**
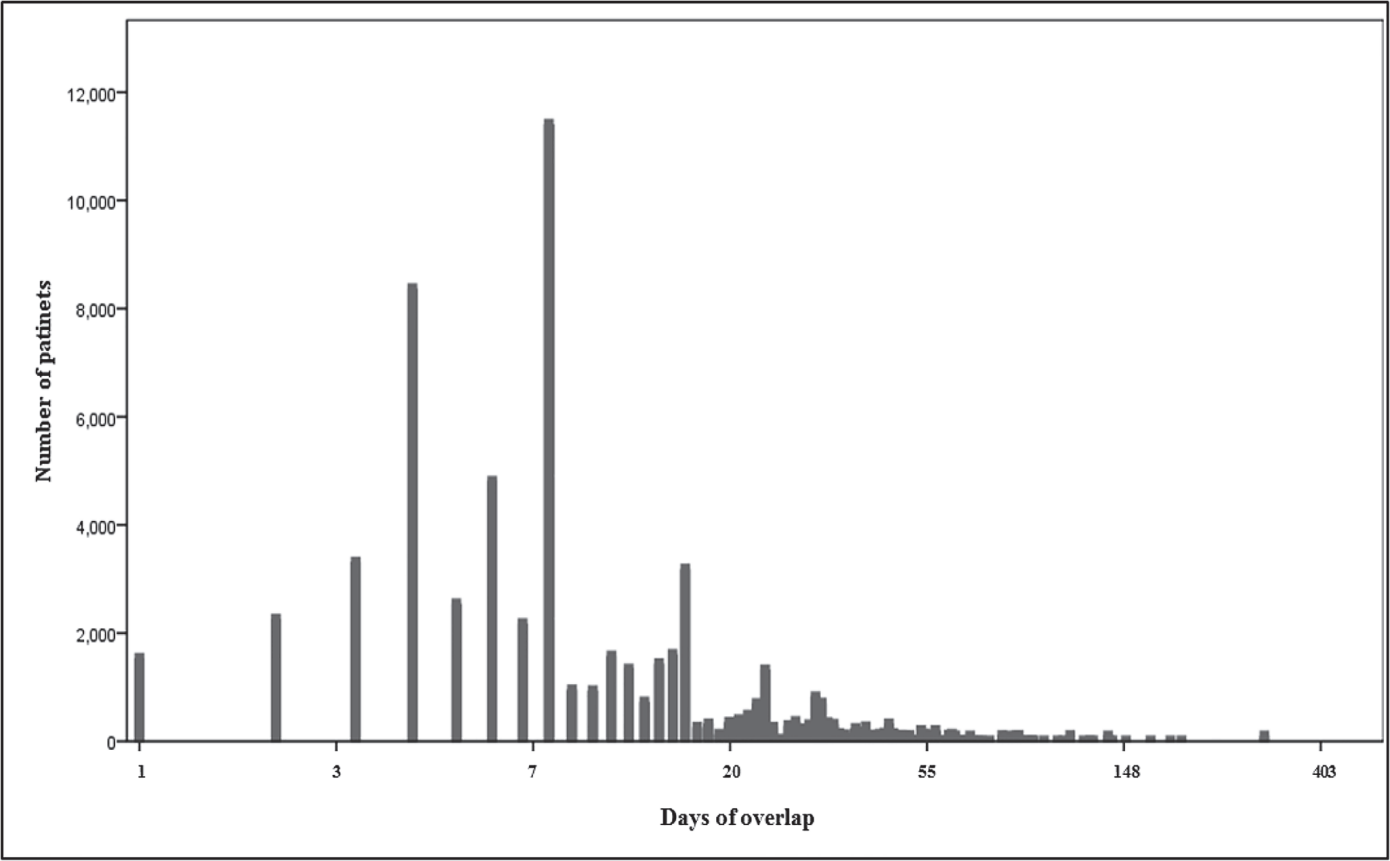
The distribution of patients by overlapping duration of CYP3A4-metabolized statins and contraindicated drugs in 2009. The days of overlap (x-axis) was expressed on a logarithmic scale. Total N = 60,254; median = 7.4; mean = 8.7; standard error = 1.0; quartile 1 = 3.9; quartile 3 = 15.1.

Among the study patients, 60,254 (2.84%) patients were co-medicated with a CYP3A4-metabolized statin and its contraindicated drug. The proportions of co-medication were 4.61% in simvastatin users, 2.17% in lovastatin users, and 1.80% in atorvastatin users, respectively. According to age group, the proportion was increased from 1.9% in the early 40s to 3.29% in the early 60s age group, then decreased to 2.05% in the oldest age group (older than 75 years). The median days of overlap was 7.4 days (Q1-Q3, 3.9–15.1) for the co-medicated patients (Fig 2).

### Co-medication according to generic name of statins

Among the co-medicated patients, 2,908 (4.8%) cases were co-prescribed, 8,000 (13.3%) cases were prescribed by the same medical institution and 51,369 (85.3%) were prescribed by different medical institutions. Co-medication in different medical institutions was the most common co-medication pattern, represented by 97.6% of co-medicated lovastatin users, 89.9% of atorvastatin users, and 80.8% of simvastatin users. The proportion of co-prescription was relatively lower in all CYP3A4-metabolized statins, with 1.2% of lovastatin users, 4.2% of atorvastatin users, and 5.5% of simvastatin users.

The median days of overlap was 7.9 days (Q1-Q3, 5.1–17.6) for atorvastatin, followed by 7.3 days (Q1-Q3, 5.6–9.3) for lovastatin and 7.1 days (Q1-Q3, 3.7–11.5) for simvastatin (Table 3).

**Table 3:** The frequency and overlapping duration of co-medicated patients according to generic name of statin in 2009

Pattern	Total (n = 60,254)			Same prescription (n = 2,908)			Same medical institution (n = 8,000)			Different medical institutions (n = 51,369)		
	Number	Overlapping days		Number (%)	Overlapping days		Number (%)	Overlapping days		Number (%)	Overlapping days	
		Mean (±SE)	Median (Q1-Q3)		Mean (±SE)	Median (Q1-Q3)		Mean (±SE)	Median (Q1-Q3)		Mean (±SE)	Median (Q1-Q3)
Simvastatin	32,085	11.0 (±0.6)	7.1 (3.7–11.5)	1,754 (5.5)	9.1 (±1.2)	7.4 (5.4–7.9)	5,569 (17.4)	6.3 (±0.6)	5.3 (2.5–7.8)	25,938 (80.8)	11.8 (±0.7)	7.1 (3.8–13.1)
Atorvastatin	27,354	16.9 (±1.4)	7.9 (5.1–17.6)	1,138 (4.2)	12.1 (±2.0)	8.2 (4.0–14.9)	2,438 (8.9)	11.7 (±1.9)	7.3 (2.6–17.4)	24,600 (89.9)	17.2 (±1.6)	7.9 (5.2–17.4)
Lovastatin	1,300	11.1 (±2.8)	7.3 (5.6–9.3)	15 (1.2)	8.0 (±0)	8.0 (8.0–8.0)	23 (1.8)	6.0 (±2.5)	4.5 (1.0–8.2)	1,269 (97.6)	11.2 (±2.9)	7.2 (5.6–9.3)
Total	60,254	13.8 (±0.7)	7.4 (3.9–15.1)	2,908 (4.8)	10.1 (±1.0)	7.5 (5.4–12.1)	8,000 (13.3)	8.0 (±0.8)	4.7 (2.3–7.9)	51,369 (85.3)	14.5 (±0.8)	7.5 (4.2–15.3)

SE = Standard error, Q1 = quartile 1, Q3 = quartile 3.

Patients included in each type of co-medication pattern were not mutually exclusive.

### Co-medication of statins with contraindicated drugs

Table 4 shows the frequency of the combinations of statins and contraindicated drugs. Because of their infrequent use, there was no patient who co-medicated with protease inhibitors. The most frequent combination was atorvastatin-itraconazole, followed by simvastatin-clarithromycin and simvastatin-itraconazole, with 25,500, 18,185, and 10,777 patients, respectively. Among patients co-medicated with atorvastatin-itraconazole, 22,654 (88.8%) patients were co-medicated by different medical institutions, 2,431 (9.5%) were co-medicated by the same medical institution and 1,131 (4.4%) were co-prescribed. Also, the proportion of co-medication associated with different medical institutions was high for other combinations.

**Table 4:** The frequency and overlapping duration of co-medicated patients according to statin-contraindicated drug combination in 2009.

	Total (Number = 60,254)				Same prescription (Number = 2,908)			Same medical institution (Number = 8,000)			Different medical institutions (Number = 51,369)		
Statin-contraindicated drug	Number	Overlapping days		Number		Overlapping days		Number	Overlapping days		Number	Overlapping days	
		Mean (±SE)	Median (Q1-Q3)			Mean (±SE)	Median (Q1-Q3)		Mean (±SE)	Median (Q1-Q3)		Mean (±SE)	Median (Q1-Q3)
ATV—ITZ	25,500	17.3 (±1.5)	8.0 (5.2–20.1)	1,131		12.2 (±2.0)	8.2 (4.0–14.9)	2,431	11.8 (±1.9)	7.3 (2.6–17.5)	22,654	17.7 (±1.7)	7.9 (5.3–20.1)
SMV—CLA	18,185	8.4 (±0.5)	6.7 (3.5–8.0)	1,338		7.5 (±0.4)	7.3 (5.2–7.7)	4,315	5.4 (±0.4)	3.7 (1.9–7.4)	13,331	9.1 (±0.7)	6.6 (3.7–9.9)
SMV—ITZ	10,777	16.1 (±1.3)	7.9 (5.4–23.1)	354		13.8 (±3.5)	7.9 (6.4–20.3)	700	10.8 (±3.5)	7.0 (1.9–15.0)	9,915	16.3 (±1.4)	7.9 (5.4–23.1)
SMV—ERY	3,762	7.0 (±1.6)	3.7 (2.9–6.4)	85		6.0 (±1.2)	4.5 (2.7–7.1)	585	7.6 (±1.6)	4.0 (3.4–10.9)	3,162	6.9 (±1.1)	3.6 (2.8–5.6)
ATV—KTZ	2,292	9.2 (±1.6)	5.6 (3.7–9.1)	8		9.0 (-)	9.0 (9.0–9.0)	8	5.0 (-)	5.0 (5.0–5.0)	2,285	9.2 (±1.6)	5.6 (3.7–9.1)
LOV—ITZ	1,177	11.6 (±3.1)	7.5 (5.8–9.5)	15		8.0 (±0)	8.0 (8.0–8.0)	23	9.0 (-)	9.0 (9.0–9.0)	1,146	11.7 (±3.2)	7.5 (5.8–9.5)
SMV—KTZ	362	6.3 (±2.2)	3.3 (1.6–3.9)	-		-	-	8	4.0 (-)	4.0 (4.0–4.0)	354	6.3 (±2.2)	3.3 (1.6–3.9)
LOV—KTZ	123	6.5 (±1.1)	5.5 (5.2–5.8)	-		-	-	-	-	-	123	6.5 (±1.1)	5.5 (5.2–5.8)

SE = Standard error, Q1 = quartile 1, Q3 = quartile 3, ATV = atorvastatin, SMV = simvastatin, LOV = lovastatin, ITZ = itraconazole, CLA = clarithromycin, KTZ = ketoconazole, ERY = erythromycin.

Patients included in each type of co-medication patterns were not mutually exclusive.

Analyzing the duration of overlap by drug combination, the longest durations were 8.0 days for atorvastatin-itraconazole and 7.9 days for simvastatin-itraconazole, and the shortest durations were 3.3 days for simvastatin-ketoconazole and 3.7 days for simvastatin-ketoconazole.

## Discussion

This study was performed to describe the co-medication patterns of statins with contraindicated drugs using a random sample from a nationally representative claims database. Among the CYP3A4-metabolized statin users, 2.84% were co-medicated with contraindicated drugs and the majority of co-medications occurred between different medical institutions. Among the statins, co-medication more commonly occurred in simvastatin users than in atorvastatin or lovastatin users. Co-medication of atorvastatin-itraconazole was the most frequent combination, followed by simvastatin-clarithromycin and simvastatin-itraconazole.

This study demonstrated that the proportion of co-medication of CYP3A4-metabolized statins with contraindicated drugs was relatively lower than that of previous studies. Bakhai et al. reported that 11% CYP3A4-metabolized statin users were co-medicated with a labeled inhibitor in a UK primary care population during 2008 [19] and approximately 9% of CYP3A4-metabolized statin users were co-medicated with labeled inhibitors in patients in a US study [20]. According to Devold et al., the rates of co-medication of potent CYP3A4 inhibitors among continuous statin users were 5.9% in 2004 and 7.0% in 2006 [21]. Due to the limited comparability of studies, it is difficult to compare the proportion of co-medication directly. However, the differences in the definition of CYP3A4 inhibitors (i.e. our study included CYP3A4 inhibitors defined as contraindicated drugs by a regulatory agency) among studies could have resulted in the lower level of co-medication found in this study. In addition, these findings may reflect the national reimbursement policy that co-prescriptions of contraindicated drugs included in the Contraindicated Drug List have been restrictively reimbursed by the Drug Utilization Review system since 2004.

The present study showed that most cases of co-medication occurred between different medical institutions. Compared with previous studies suggesting that the co-medication typically occurred by the same prescriber, co-medication by different medical institutions was found to be relatively high in this study. According to Stang et al., 71.2% of cases of co-medication of a statin and contraindicated drug originated from the same prescriber [22], but a Norwegian study showed that 53.5% of cases of co-medication of short-term CYP3A4 inhibitors and a statin were prescribed by different physicians [21]. With the impact of a national policy that restricts co-prescribing of contraindicated drugs, the proportion of co-prescription could have been low compared to other countries. However, we observed that the proportion of co-medication associated with different medical institutions was high. This implies that a more effective management system to control for co-medication between different medical institutions is needed. The inspection of co-medication of contraindicated drugs by the concurrent Drug Utilization Review system, which was launched in 2010 [23], expanded the scope to medical services between different medical institutions.

The proportion of co-medication was greater in simvastatin users than lovastatin and atorvastatin users. Though the relatively numerous contraindicated drugs of simvastatin and the delayed notification date of atorvastatin-itraconazole could have affected our findings, our results showed evidence of efforts to manage the co-medication of simvastatin users. It was noted earlier that the interaction risk of each CYP3A4-metabolized statin is not identical. Simvastatin and lovastatin are more sensitive than atorvastatin. The area under the plasma concentration-time curve (AUC) of simvastatin and lovastatin have been shown to increase up to about 20-fold with concomitant use of strong CYP3A4 inhibitors. However, the AUC of atorvastatin with concomitant use of strong CYP3A4 inhibitors has been shown to increase by up to about 5-fold [11,24]. Previous research has demonstrated that the ratio of adverse event reporting of rhabdomyolysis associated with simvastatin stratified by concomitant use of CYP3A4 inhibitor was 6.3 [25]. Another study analyzing adverse reports showed 77% of reports of rhabdomyolysis associated with simvastatin and 44% of such reports associated with atorvastatin involved interacting medicines [8].

Despite being contra-indicated by the KMHW from 2004, co-medication of atorvastatin-itraconazole, simvastatin-clarithromycin, and simvastatin-itraconazole and were most commonly identified among the combinations. Mostly, itraconazole was prescribed to treat dermatophytosis or candidiasis, and the most common main diagnosis for prescribing clarithromycin was acute bronchitis. When co-administered simvastatin with itraconazole or clarithromycin, the AUC of simvastatin increased up to 20-fold or 12-fold, respectively [11]. Case reports have noted that rhabdomyolysis occurred due to the interaction of simvastatin-itraconazole or clarithromycin [26,27] Also, with too a lesser extent, itraconazole significantly increased the AUC of atorvastatin to 3.2 fold [28]. Ballantyne et al. recommended considering the discontinuation of a statin during a short course of macrolide antibiotics [29] and national guidelines for managing drug-drug interaction in Netherlands proposed stopping statin use or changing to pravastatin or rosuvastatin during use of antimycotics or macrolides [30].

Our study had several strengths. First, the study population represented Korean patients by using the national health insurance claims database, which covers nearly the entire Korean population. Second, because the HIRA database included comprehensive information on drug use (i.e. generic name, prescription date, prescription duration) and medical institution, this study was able to provide detailed and precise information on co-medication patterns. The use of a claims database minimized the possibility of recall bias for drug use. Also, the overall use of a statin and contraindicated drug (i.e. antifungals and macrolides for systematic use, and protease inhibitors) was investigated completely because the study drugs were all prescription drugs.

The limitations of this study included the following: As with other studies using a claims database of prescriptions, we could not determine the direct drug exposure. Moreover, it was difficult to identify whether patients temporarily discontinued statin use while being treated with contraindicated drugs according based on the advice of health care professionals. We focused on the prevalence and pattern of co-medication, however, co-medication could not always translate into clinical outcome. To evaluate the risk of unfavorable clinical outcome of co-medication is needed.

In this study, only officially contraindicated drugs were included to focus on co-medication patterns with contraindicated drugs. Gemfibrozil was also known for drug interaction with statin on membrane transporter that increasing risk of myopathy [31–34]. Among total patients who were prescribed CYP3A4-metabolized statins, 138 (0.0065%) were co-medicated with gemfibrozil, and 94.2% were co-medicated in same prescription. Co-medication pattern of gemfibrozil was distinct from officially contraindicated drug. Further research is required to examine the broader scope of drug interaction of statin.

In summary, the results of this study suggest that more efforts will be needed for managing co-medication between different medical institutions. A computerized clinical decision support system has been implemented nationally to reduce contraindicated drug use and to prevent overlapping use of the same drug since December 2010 in Korea [23]. Along with this technical support, additional education for health care professionals on managing drug interactions such as suggestion of drugs in same therapeutic class without drug interaction is also needed.
